# Artemin growth factor increases nicotinic cholinergic receptor subunit expression and activity in nociceptive sensory neurons

**DOI:** 10.1186/1744-8069-10-31

**Published:** 2014-05-22

**Authors:** Kathryn M Albers, Xiu Lin Zhang, Charlotte M Diges, Erica S Schwartz, Charles I Yang, Brian M Davis, Michael S Gold

**Affiliations:** 1 Center for Pain Research, University of Pittsburgh, Pittsburgh, PA 15261, USA; 2 Department of Neurobiology, Biomedical Sciences Town, Room E1454, University of Pittsburgh School of Medicine, 200 Lothrop St., Pittsburgh, PA 15261, USA; 3 Department of Anesthesiology, University of Pittsburgh, Pittsburgh, PA 15261, USA

**Keywords:** Artemin, Growth factor, Nicotinic receptor, Inflammation, Skin

## Abstract

**Background:**

Artemin (Artn), a member of the glial cell line-derived growth factor (GDNF) family, supports the development and function of a subpopulation of peptidergic, TRPV1-positive sensory neurons. Artn (enovin, neublastin) is elevated in inflamed tissue and its injection in skin causes transient thermal hyperalgesia. A genome wide expression analysis of trigeminal ganglia of mice that overexpress Artn in the skin (ART-OE mice) showed elevation in nicotinic acetylcholine receptor (nAChR) subunits, suggesting these ion channels contribute to Artn-induced sensitivity. Here we have used gene expression, immunolabeling, patch clamp electrophysiology and behavioral testing assays to investigate the link between Artn, nicotinic subunit expression and thermal hypersensitivity.

**Results:**

Reverse transcriptase-PCR validation showed increased levels of mRNAs encoding the nAChR subunits α3 (13.3-fold), β3 (4-fold) and β4 (7.7-fold) in trigeminal ganglia and α3 (4-fold) and β4 (2.8-fold) in dorsal root ganglia (DRG) of ART-OE mice. Sensory ganglia of ART-OE mice had increased immunoreactivity for nAChRα3 and exhibited increased overlap in labeling with GFRα3-positive neurons. Patch clamp analysis of back-labeled cutaneous afferents showed that while the majority of nicotine-evoked currents in DRG neurons had biophysical and pharmacological properties of α7-subunit containing nAChRs, the Artn-induced increase in α3 and β4 subunits resulted in functional channels. Behavioral analysis of ART-OE and wildtype mice showed that Artn-induced thermal hyperalgesia can be blocked by mecamylamine or hexamethonium. Complete Freund’s adjuvant (CFA) inflammation of paw skin, which causes an increase in Artn in the skin, also increased the level of nAChR mRNAs in DRG. Finally, the increase in nAChRs transcription was not dependent on the Artn-induced increase in TRPV1 or TRPA1 in ART-OE mice since nAChRs were elevated in ganglia of TRPV1/TRPA1 double knockout mice.

**Conclusions:**

These findings suggest that Artn regulates the expression and composition of nAChRs in GFRα3 nociceptors and that these changes contribute to the thermal hypersensitivity that develops in response to Artn injection and perhaps to inflammation.

## Background

The prolonged release and activation of cellular signaling pathways by growth factors and cytokines contributes to the sensitization of primary afferents that transmit pain [1]. The neurotrophic factors, nerve growth factor (NGF) and artemin (Artn), may have a particularly important role in this process. Receptors for each of these growth factors, where Artn binds the receptors GFRα3 and Ret and NGF binds the receptors p75 and trkA, are expressed by a subset of peptidergic, TRPV1-positive C-fibers. GFRα3/trkA afferents comprise 20-25% of the trigeminal and dorsal root ganglia (DRG), are small to medium in size, are heat responsive and terminate in spinal cord lamina 1 [2,3]. Approximately 80% of these fibers are CGRP positive and nearly all (95-99%) express the algogenic receptors TRPV1 and TRPA1.

Artn expression is elevated in several inflammatory conditions, which include pancreatic disease, atopic dermatitis and following UV-irradiation of the skin [4,5]. In rodents, experimentally induced inflammation of the skin increases the level of Artn mRNA and this increase parallels the time course of thermal hyperalgesia [6,7]. Other studies have shown that injection of anti-Artn antibodies provides a partial reversal of complete Freund’s adjuvant (CFA)-induced mechanical hypersensitivity [7]. In addition, cultured DRG neurons grown in the presence of Artn exhibit enhanced capsaicin-evoked Ca^2+^ influx and release of CGRP [8]. Finally, mice that overexpress Artn in skin keratinocytes (ART-OE mice) and wildtype (WT) mice injected with Artn exhibit increased sensitivity to chemical (capsaicin/mustard oil) and thermal stimuli. These behavioral changes likely reflect, at least in part, enhanced expression of TRPV1 and TRPA1 in the DRG of these mice [6,9,10].

To better understand how Artn affects sensory neuron properties and its role in inflammatory conditions, we carried out transcriptional profiling of trigeminal ganglia of the Artn overexpressing mice. Interestingly, a significant increase in the nicotinic acetylcholine receptor (nAChR) subunit genes Chrnα3, Chrnβ3, and Chrnβ4 was found, suggesting nAChR’s contribute to changes in sensitivity exhibited by these mice. Nicotinic AChRs are excitatory, pentameric ion channels expressed throughout the central and peripheral nervous system that are activated by acetylcholine [11]. The presence of nAChR subunits in sensory neurons has been well documented by binding, expression and functional assays [12-15]. Electrophysiological and anatomical evidence also place nAChRs primarily in capsaicin-sensitive, peptidergic Aδ and C fiber afferents [15,16], suggesting a role for these channels in nociceptive signaling [17,18]. Consistent with such a role, application of acetylcholine (ACh), nicotine or other cholinergic agonists applied to the skin, arterial, tongue, nasal or ocular surfaces elicits burning pain [19-24].

Here we show that enhancing the level of skin-derived Artn increases the expression and activity of nAChRs in cutaneous neurons and that CFA-induced tissue inflammation does so as well. In addition, peripheral administration of nAChR antagonists was found to attenuate Artn-induced thermal hypersensitivity in ART-OE mice and mice acutely injected with Artn, suggesting both short- and long-term consequences of Artn signaling on cholinergic activity. These findings suggest a new mechanism for the regulation of noxious sensation that links elevation of growth factors with enhanced signaling in the nicotinic cholinergic system.

## Results

### Artn increases nAChR subunit expression in sensory ganglia

Transgenic mice that overexpress Artn in the skin under control of the K14 keratin promoter exhibit thermal and chemical hypersensitivity [9,10]. To identify changes in gene expression that could contribute to this hypersensitivity, transcriptional profiling of trigeminal ganglia (TG) of wildtype (WT) and ART-OE mice was carried out. Interestingly, a marked increase in mRNAs encoding the α3, β3 and β4 subunits of the nicotinic acetylcholine receptor was found in ART-OE ganglia (Table 1). SYBR green real time-PCR (RT-PCR) assays used to validate these increases show that α3, β3 and β4 are increased in TG of ART-OE mice but only α3 and β4 were increased in lumbar DRG (L3-L5) (Table 1). This difference in expression between the TG and DRG may relate to the different innervation targets in the respective peripheral fields. The greatest increase in mRNA was measured for α3, which in the TG was elevated ~13-fold. RT-PCR assays also showed a 1.38-fold decrease in the α2 transcript level in the DRG.

**Table 1:** Expression of nAChR subunits in ART-OE trigeminal (TG) and lumbar DRG

**nAChR subunit**	**Fold change microrarray**	**Fold change RT-PCR**	
	**TG**	**TG**	**DRG**
α2	1.06	ND	−1.38*
α3	4.84	13.35*	3.98*
α4	0.67, 1.02	NC	
α5	0.9	NC	
α6	1.38	NC	
α7	1.05	NC	
α9	1.06	NC	
β2	0.92, 0.96, 1.01	NC	
β3	3.13, 3.2, 2.06	4.08*	NC
β4	1.56	7.68*	2.82*

Microarray data comparing ART-OE (n = 3) and WT (n = 3) mice was validated using SYBR green qRT-PCR assay. Multiple values in array data indicate number of times gene was represented on array. RT-PCR assays analyzed RNA isolated from 3–6 mice. NC = not changed, ND = not done. Asterisk indicates p <0.05.

**Figure 1:**
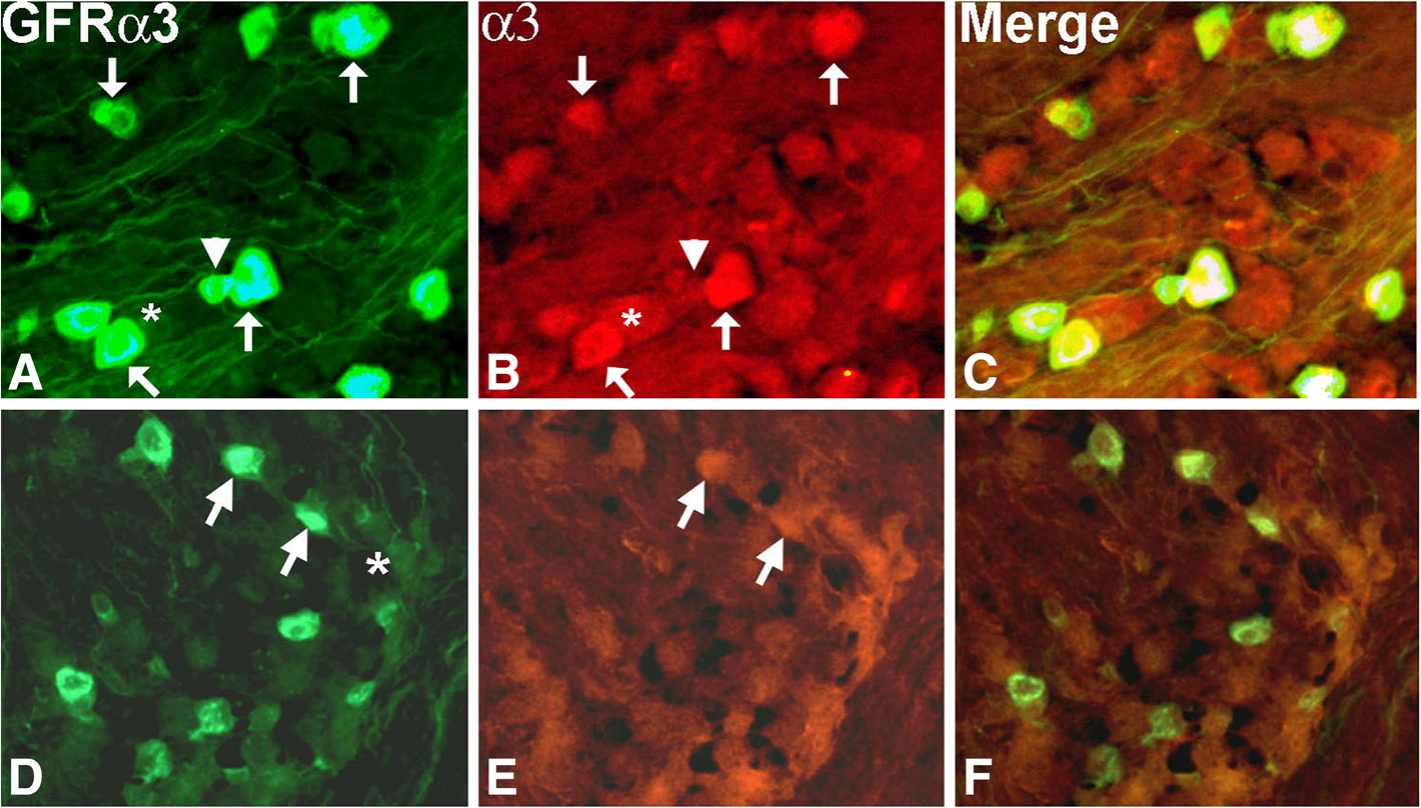
**Immunolabeling shows nAChRα3 is expressed in GFRα3-neurons.** Labeling of nAChRα3-positive neurons in ART-OE ganglia **(A-C)** is more intense compared to WT ganglia **(D-F)**. Quantitative analysis showed the percentage of GFRα3 neurons that express nAChRα3 is greater in ganglia of ART-OE mice (46%) compared to WT mice (9.7%) (p < 0.05%, Student’s t test). Arrows indicate GFRα3/nAChRα3 positive neurons; asterisks mark nAChRα3 neurons that are not GFRα3 positive; arrowhead shows GFRα3 neuron that is nAChRα3 negative.

Because Artn specifically binds and activates signaling pathways downstream of GFRα3, i.e., it does not activate GFRα1 and GFRα2 [25], a prediction was that changes in nAChRs would be associated with GFRα3-positive neurons. We therefore used immunolabeling with tyramide amplification to assess the co-localization of nAChRα3 and GFRα3 proteins in the trigeminal ganglia of ART-OE and WT mice (Figure 1). We analyzed expression of nAChRα3 because it showed the greatest increase in the TG of ART-OE mice. Immunolabeling with GFRα3/nAChRα3 antibodies showed a greater intensity of nAChRα3 labeling in ART-OE ganglia relative to wildtype control ganglia. Quantitative analysis of the overlap in GFRα3/nAChRα3 was carried out and showed a 4.74-fold increase in co-labeled neurons in ganglia of ART-OE mice relative to ganglia of wildtype mice (n = 3 mice/group, p < 0.05).

### Genetic knockout of Artn/GFRα3 signaling reduces nAChR expression

To further assess the link between Artn and nAChR subunit expression, we used real time reverse transcriptase assays to measure the relative level of mRNAs encoding nAChR subunits in ganglia of Artn- and GFRα3-knockout mice. ART-KO and GFRα3-KO mice, which are viable and reported to have no loss in sensory neurons [26-28], had reduced levels of nAChR α3 and β3 mRNAs (1.3-2.6-fold), but no change in β4 in sensory ganglia (Table 2).

**Table 2:** **nAChR subunit and TRP ion channel mRNA level is reduced in sensory ganglia of *Artn *** **and *GFRα3 *** **knockout mice**

**Mouse strain/gene assayed**	**Fold change trigeminal**	**Fold change DRG**
ART-KO		
GFRα3	−1.4*	−1.23*
Ret	NC	NC
TRPV1	−1.28*	−1.30*
TRPA1	−1.87*	NC
AChRα3	−2.12*	−1.27*
AChRβ3	−1.58*	−1.54*
AChRβ4	NC	NC
GFRα3-KO		
TRPV1	−1.56*	−1.51*
TRPA1	NC	−1.30*
AChRα3	−2.61*	−1.27*
AChRβ3	−1.15*	−1.36*
AChRβ4	NC	NC

Fold change in mRNAs using SYBR green RT-PCR analysis of lumbar (L2/3/4) DRGs or TG. N = 3–6 animals per group. NC, not changed. *P < 0.05.

### Neurturin signaling also changes nAChR subunit transcription

To determine if other growth factors over-expressed in the skin alter nAChR expression we assayed the relative level of nAChR mRNAs in TG and DRG of mice that overexpress the neurotrophin NT3 (NT3-OE) [29] or neurturin (Nrtn-OE) [30]. NT3-OE ganglia showed no change in either α3 or β3 (not shown) whereas ganglia of Nrtn-OE mice had increased α3 (1.37-fold), α6 (1.54-fold) and β2 (1.27-fold) and decreased β4 (1.48-fold) and α5 (1.39-fold) (n = 3-4 per group, p < 0.05). Thus, Nrtn also increased α3 but showed changes in several other subunits that are distinct from those caused by Artn.

### Artn overexpression in skin causes functional changes in nAChRs in cutaneous afferents

**Figure 2:**
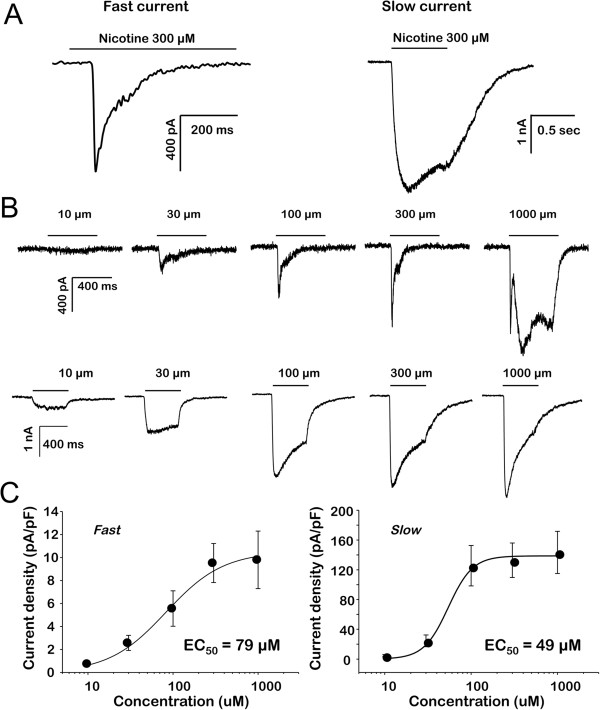
**Nicotine evoked currents in acutely dissociated DRG neurons.** Nicotine was applied for 500 ms to neurons held at −60 mV via a fast application system. **A**. Two types of currents were detected in mouse DRG neurons that were distinguishable based on differences in activation and inactivation kinetics: A fast current (left) that activated and inactivated rapidly and a slow current (right) that activated and inactivated more slowly. **B**. Fast (top traces) and slow (bottom traces) currents were activated by nicotine over a comparable concentration range. Fast and slow currents were evoked from the same neuron in response to increasing concentrations of nicotine. A third even more slowly activating current (top trace, right side) was evoked at higher (1000 μM) concentrations of nicotine and was present in most neurons with fast current as well as a significant number of neurons without either fast or slow current. **C**. Concentration response data from individual neurons with fast (n = 26) or slow (n = 16) current were pooled and fitted with a Hill equation to estimate the maximal evoked current and the concentration at which a current 50% of maximal was evoked (EC50).

**Figure 3:**
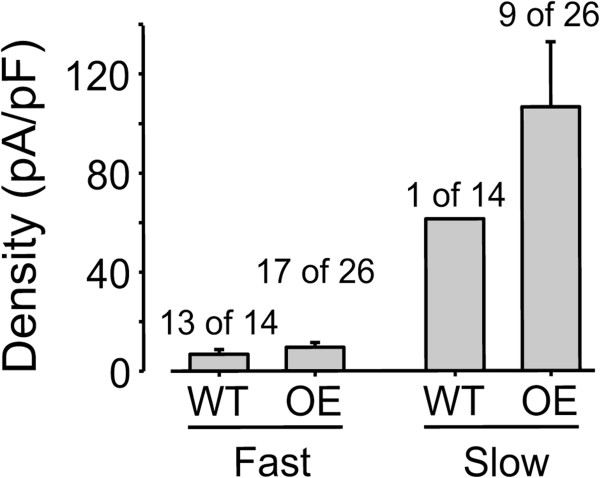
**Nicotine-evoked current density.** Current density was determined by dividing the peak-evoked current in response to 300 μM nicotine by the membrane capacitance. Pooled data from neurons with fast and slow current indicate that the slow current density is significantly greater than that of the fast current. The proportion of neurons from wild type (WT) and ART-OE (OE) mice with evoked current that was either fast or slow is indicated above each bar.

While an increase in nAChR subunit expression should be associated with an increase in functional receptors in cutaneous afferents, it was important to confirm this expectation. We therefore assessed changes in nAChR properties using patch-clamp electrophysiology. We compared nicotine-evoked currents in DiI-backlabeled cutaneous afferents in DRG of wildtype and ART-OE mice. Two major current types were observed that were distinguished by rates of activation and inactivation; a fast current that activated and inactivated rapidly and a slow current that activated and inactivated slowly (Figure 2A). The nicotine concentration range over which fast and slow currents were activated were comparable, with EC50’s of 79 and 49 μM, respectively (Figure 2B and C). Slow currents were, on average, significantly larger than fast currents with a peak current density for the slow current almost an order of magnitude greater than that for the fast current (Figure 2C). Nicotine-evoked currents were only detected in a minority (14 of 74) of cutaneous neurons from WT mice, and the majority of these (13 of 14) were fast currents (Figure 3, Table 3). In contrast, while the overall increase in the proportion of cutaneous neurons from ART-OE mice with nicotine-evoked currents (29 of 89) was not significant, the increase in the proportion of neurons from ART-OE mice with slow current (9 of 89) was significant (p < 0.05, Fisher Exact test).

**Table 3:** Properties of nicotinic responder neurons from WT (n = 6) and ART-OE (n = 7) mice defined by IB4 lectin binding, size and capsaicin sensitivity

**Strain**	**Total cells**	**IB4+**	**IB4-**	**Small**	**Medium**	**Cap pos**	**Cap neg**
**Wildtype**	74	29	39	61	13	11	36
*With current*	14	0	14	5	9	2	5
*No current*	60	29	25	56	4	9	31
*Fast current*	13	0	13	5	8	-	-
*Slow current*	1	0	1	1	0	-	-
**ART-OE**	89	13	28	71	18	13	27
*With current*	29	1	17	19	10	-	-
*No current*	60	12	19	52	8	-	-
*Fast current*	20	1	14	14	6	-	-
*Slow current*	9	0	4	6	3	-	-

**Figure 4:**
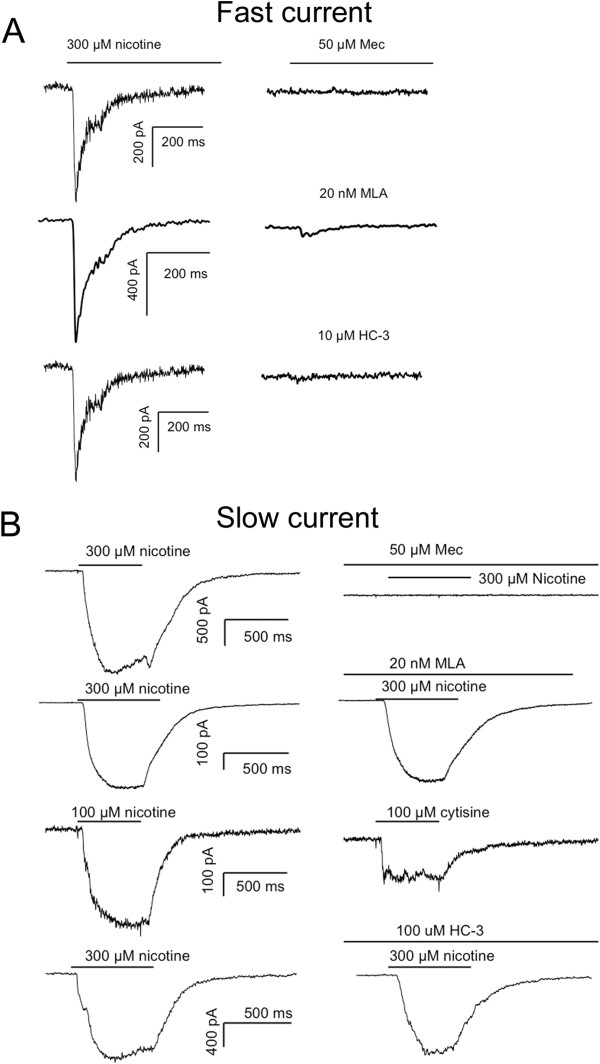
**Pharmacological characterization of nicotine evoked currents. A**. The fast current was blocked by the non-specific nicotinic receptor antagonist mecamylamine (Mec, top trace), the α7-subunit antagonist MLA (middle trace) and the TRPA1 “selective” antagonist HC-30031 (HC, bottom traces). Currents are from three different neurons in response to nicotine applied with a 5 min inter-stimulus interval before and after the application of antagonist. **B**. The slow current was also blocked by Mec (top row) but was resistant to MLA (second row) and activated by the α4β3 agonist cytisine (third row). The slow current was also resistant to HC-30031. As in **A**, current in each row was evoked from a different neuron. All data in **A** and **B** were from ART-OE mice, but comparable data for the fast current were obtained from WT mice.

Because of recent findings that nicotine activates TRPA1 [31], and our own data showing that Artn increases TRPA1 expression [10], we performed several additional experiments to further define the nature of the nicotine-evoked currents in cutaneous neurons. First, we performed concentration-response analysis of both fast and slow currents in cutaneous neurons (Figure 2B, C). While the potency of nicotine-induced activation of inward current in DRG neurons was comparable to that previously reported for the activation of TRPA1, the kinetics of activation of the currents we describe are considerably faster than those previously reported for TRPA1, even at relatively low concentrations (Figure 2B). Of note, a second, even more slowly activating current (i.e., see Figure 2B), most clearly evident at 1 mM nicotine, was detected in ~30% and ~80% neurons studied from WT and ART-OE mice, respectively, and may reflect the TRPA1 current previously described. Second, we characterized the pharmacological properties of the fast and slow nicotine evoked currents (Figure 4). Both current types were completely blocked by the non-subtype selective antagonist mecamylamine (50 μM). Because the biophysical properties of the fast current were comparable to those of α7-subunit containing nAChRs, we assessed the actions of the α7-selective antagonist, MLA (20 nM). MLA completely blocked the fast current (Figure 4A), but had no detectable influence on the slow current (Figure 4B). Because the heterologous expression of α4β3 subunit containing nAChRs results in a current with biophysical properties of the slow current in mouse cutaneous neurons [32], we assessed the impact of the α4β3- agonist cytisine (100 μM). Cytisine activated a slowly activating current in 4 of 4 neurons tested with a slow current, but 0 of 4 neurons tested with a fast current (p < 0.05). Finally, to rule out the possibility that the nicotine-evoked currents involved the activation of TRPA1, we assessed the impact of the TRPA1 antagonist HC-030031 on nicotine evoked currents. Surprisingly, HC-030031 completely blocked the fast current (n = 4, Figure 4A) but importantly, had no detectable influence on the slow current (Figure 4B). A second TRPA1 antagonist was tested on the fast current, A-967079, but this too completely blocked the fast current (n = 3; data not shown). While the results with these TRPA1 antagonists raise several interesting questions, as this was not the focus of the present study, these questions were not pursued here. Third, because GFRα3 is present in a subpopulation of putative nociceptive, peptidergic sensory neurons, we sought to determine whether there was a differential distribution of fast and slow currents among cutaneous neurons. However, because of evidence that DRG neurons hypertrophy in the ART-OE mouse [10], we did not include cell body size as a criterion with which to identify putative nociceptive afferents. Rather, results obtained with both small and medium neurons were pooled. This analysis, summarized in Table 3, indicates that both fast and slow currents are largely restricted to a subpopulation of IB4 negative cutaneous neurons.

### nAChR antagonists block Artn-induced thermal hyperalgesia

**Figure 5:**
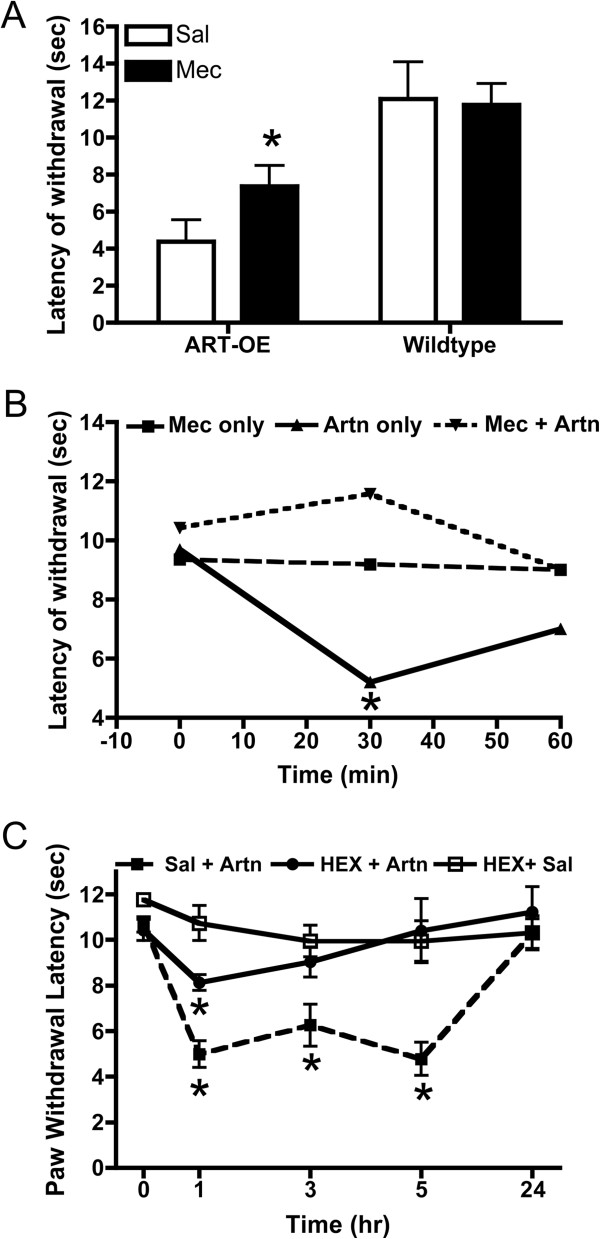
**nAChR antagonists block Artn-induced heat hyperalgesia. A**. WT (n = 4) and ART-OE (n = 6) mice were injected with either 10 μl saline (right foot) or 10 μl Mec (left foot, 1 mg/kg) and latencies of withdrawal measured for each foot 60 min post injection. As previously reported [10], ART-OE mice exhibit lower heat thresholds compared with WT mice (compare saline treated groups). Mec partially blocks heat hyperalgesia in ART-OE mice (p < 0.05, ANOVA) and did not affect thermal sensitivity in WT mice. **B**. Footpads of WT mice were injected with 1 mg/kg Mec or saline 30 min prior to injection of Artn (200 ng) or saline. Mec injection had no effect on thermal sensitivity. Artn injection alone increased heat hyperalgesia whereas Mec + Artn blocked hyperalgesia. **C**. Mice (n = 6) were injected in the left footpad with saline (10 μl) 30 min prior to Artn injection (200 ng). Other mice (n = 6) were injected with HEX (10 μl, 1 mg/kg) followed by Artn. A third set (n = 6) were injected with HEX followed by saline. HEX alone did not affect thermal sensitivity. HEX blocked thermal sensitivity caused by Artn at 1 h, 3 h and 5 h post injection. Measures were made in a blinded manner. Asterisks indicate p < 0.05.

Injection of Artn causes thermal hypersensitivity that lasts over 24 h [6,7]. To determine if activation of nAChRs contributes to this transient sensitivity we examined the effect of nAChR antagonists on Artn-induced behavioral hyperalgesia. Footpads of WT mice were injected with either Artn alone, the broad-spectrum nAChR antagonist mecamylamine [33], or both compounds. In WT mice Artn-induced a heat hypersensitivity that was blocked by mecamylamine as indicated by normalization of foot withdrawal latencies (Figure 5A). Mecamylamine injection was found to also block the inherent thermal hypersensitivity exhibited by ART-OE mice (Figure 5B).

Because mecamylamine can cross the blood brain barrier and has been shown to block TRPA1 channel activity [31] we determined if hexamethonium, a peripherally restricted (at low concentration) nAChR antagonist, also blocks Artn-induced thermal sensitivity. Pre-injection of hexamethonium in footpad skin 30 minutes prior to Artn injection significantly inhibited Artn-induced heat hypersensitivity for up to 5 h (Figure 5C). Thus, blocking nAChR signaling inhibits behavioral thermal hypersensitivity caused by Artn injection into the skin.

### Peripheral inflammation increases nAChR α3 and β4 gene expression in DRG

**Figure 6:**
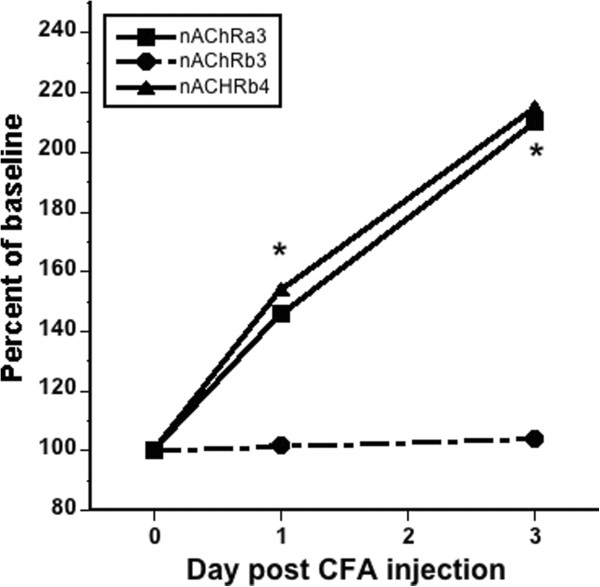
**CFA-induced skin inflammation increases nAChR mRNA levels in DRG.** The percent of baseline expression of nAChR mRNAs in lumbar DRG of uninjected mice (n = 5) and CFA injected mice (n = 6) was compared using SYBR green qRT-PCR at 1 d and 3 d post injection of CFA into footpads of C57BL/6 J mice. Percent change normalized to gapdh is plotted. Asterisks indicate significant change in β4 at day 1 and α3 at day 3 (two-way ANOVA with Bonferroni posttests with p < 0.05).

Inflammation of the skin induced by CFA injection increases the level of Artn mRNA [6,7]. Based on the above results, the elevation in Artn predicts an increase in nAChR mRNA level in response to CFA. RT-PCR analysis of RNA isolated from DRG of CFA injected mice does show increases in α3 and β4 mRNAs (1.5-fold at 1d and 2.1-fold at 3d post CFA, respectively) (Figure 6). No change in β3 mRNA was detected at these time points, which is consistent with the finding that β3 is unchanged in DRG of ART-OE mice (Table 1).

### Increased nAChR expression by Artn is independent of TRPV1 or TRPA1 activity

The enhanced expression of nAChR subunits and the ion channels TRPV1 and TRPA1 in ganglia of ART-OE mice raises the possibility of regulatory interactions between these receptor types. For example, increased expression and activity of TRP channels may alter cell signaling pathways and lead to increased expression of nAChRs or vice versa. To investigate this possibility we used RT-PCR to measure the relative level of nAChR mRNAs in DRG of hybrid ART-OE/TRPV1^−/−^/TRPA1^−/−^ mice, generated by crossing ART-OE mice with TRPV1/TRPA1 double knockout mice (Table 4). Results show that even in the absence of TRPV1 and TRPA1 function, nAChR gene expression is elevated in DRG of ART-OE mice. Thus, the increase in nAChRs in response to Artn is not dependent on TRP signaling.

**Table 4:** Artn-induced changes in nAChR subunit transcription occur in the absence of TRPV1 and TRPA1 activity

**Mouse strain**	**nAChRα3**		**nAChRβ4**		**nAChRβ3**	
	***TG***	***DRG***	***TG***	***DRG***	***TG***	***DRG***
ART-OE	13.35*	3.99*	7.68*	2.83*	4.08*	NC
ART-OE x TRPV1^−/−^/TRPA1^−/−^	12.42*	2.36*	6.69*	2.22*	3.53*	NC

Values are expressed as fold-change relative to WT ganglia. NC, not changed. Asterisk indicates significant difference with p < 0.05.

## Discussion

In this study we report that Artn, a growth factor that is elevated in inflamed tissue, increases nAChR subunit transcription in a subunit specific manner. This increase correlated with functional changes in nAChR response properties in DRG neurons and contributed to Artn-induced thermal hypersensitivity. Changes in nAChR subunit expression and nicotine-evoked current primarily affected the peptidergic, GFRα3-positive DRG neuron subpopulation, which is consistent with findings of Flores and colleagues [14], who showed using *in situ* hybridization that α3 and β4 are co-expressed in medium-diameter neurons. This observation is also consistent with Spies et al. [34] who reported small- to medium-sized neurons express the α3 nAChR subunit. In addition, since ~95% of GFRα3 neurons are TRPV1-positive, the overlap in GFRα3/nAChRα3 labeling is consistent with work of Dussor et al. [12], who showed overlap of TRPV1 and nAChRα3 mRNAs, and studies of Roberts et al., and Rau et al., who used patch clamp analysis of isolated neurons to show functional overlap of nAChR and TRPV1 activity [15,16].

Functional analysis of cutaneous DRG neurons from wildtype and ART-OE mice revealed three populations of neurons based on their response to nicotine: those with no current (at least to nicotine < 300 μM), and those exhibiting either a fast or slow current. The fast current had biophysical properties consistent with those of α7-subunit containing nAChRs while properties of the slow current were comparable to α3/β4-subunit containing nAChRs [35]. With the exception of the observation that fast currents were blocked by two TRPA1 antagonists, the pharmacological sensitivity of the fast and slow currents were consistent with those of α7- and α3/β4-subunit containing nAChRs, respectively: fast currents were blocked by the α7-selective antagonist MLA and slow currents were activated by the α3/β4 agonist cytisine. Consistent with the changes in subunit expression, the percentage of neurons from ART-OE and WT mice with a fast current was equivalent, whereas slow currents were significantly more prevalent among neurons from ART-OE mice (9 of 89 neurons) compared to WT mice (1 of 74 neurons).

The distribution of fast and slow currents was also consistent with their expression in neurons that express GFRα3: both current types were found only in IB4-negative neurons. This observation raises the possibility that changes in nAChR gene expression are directly regulated by Artn activation of GFRα3 signaling pathways. While another possibility is that this shift reflects increased survival of neurons exhibiting slow current in ART-OE ganglia, our preliminary data from rat suggesting that there is an increase in the proportion of neurons with slow current in the presence of persistent inflammation argues against this possibility [36].

Changes in nAChR subunit expression also were measured in ganglia of Nrtn-OE mice. Although there was some overlap in the pattern of subunit expression, Nrtn caused a change in nAChRs distinct from that detected in ganglia of ART-OE mice. This suggests that signaling pathways activated downstream of each GFRα receptor can modulate changes in nAChR expression. Furthermore, there is evidence that other growth factors regulate nAChR subunit expression: neuregulin 1 signaling through the ErbB kinase receptor increases nAChRs in developing skeletal muscle [37] and increases α3 and β4 mRNA in pelvic ganglion neurons [38]. In addition, cultured PC12 cells treated with NGF exhibit increased nicotinic current density and mRNAs that encode nAChRs α3, α5, α7 β2, β4 [39]. With respect to NGF, it is of interest to note that most GFRα3-positive neurons express receptors for NGF, a well-characterized inflammatory mediator. This co-responsiveness is likely to have functional significance; in previous studies we showed a synergistic interaction between Artn and NGF, where a single injection of each factor produced a transient (24 h) heat hyperalgesia but coinjection of Artn and NGF produced hyperalgesia that lasted for more than 6 days [6]. That both NGF and Artn can increase in inflamed tissue and both can modulate nAChR expression suggests the complex interplay between these factors that leads to long-term activation of the GFRα3-positive population could involve changes in cholinergic signaling.

For Artn, identifying which of the numerous GFR signaling cascades such as src, PKA, PLC and MAPKs [40] that could potentially influence nAChR gene transcription will require further study. Nevertheless, the fact that heterogeneity in nAChR subunit composition is largely responsible for heterogeneity in nAChR signaling suggests the subpopulation-specific expression of nAChRs could contribute to the unique functional properties exhibited by GFR- and trk-positive neuronal subtypes [10,30,41].

That mecamylamine and hexamethonium reduce thermal sensitivity in ART-OE mice and WT mice injected with Artn, suggest that nAChR activity contributes to thermal sensitivity. Preliminary data suggest ACh levels are elevated in CFA inflamed skin (at 3 d post CFA injection a 1.46-fold increase in ACh was measured in inflamed skin relative to skin of naïve mice (CFA 8.9 μg/ml vs. naive 4.7 μg/ml supernatant; n = 3 CFA mice and 2 naïve mice)). ACh is also elevated in human skin conditions such as atopic dermatitis [42]. The increase in ACh raises the possibility that endogenous ACh could contribute to persistent depolarization of nociceptive afferents. Persistent depolarization would require the slowly inactivating α3β4-type channels increased by Artn and could account for the increased sensitivity to thermal stimuli [10]. While the fast currents should also contribute to activation of nociceptive afferents, the persistent α3β4-type channels are also likely to contribute to the aversive responses elicited by ACh, nicotine and other cholinergic agonists applied to the skin, arterial, tongue, nasal, or ocular surfaces [21-24]. Consistent with the suggestion that nicotine can generate a persistent depolarizing drive in nociceptive afferents, in a rat skin-saphenous nerve preparation, nicotine not only induced a dose-dependent activation of C-fibers, but sensitized them to heat stimulation [43]. Such a mechanism could also account for the well established link between smoking and the severity of chronic pain conditions, such as lower back pain [44], diabetic neuropathy [45,46], fibromyalgia and pancreatitis [47]. The presence of nAChRs on peptidergic afferents also suggests that these channels contribute to the regulation of the efferent function of these afferents, e.g., transdermal iontophoresis of ACh in humans causes flare by neuropeptide release which can be blocked by hexamethonium [48].

Previous studies of ART-OE mice have shown elevation of TRPV1 and TRPA1 mRNAs in GFRα3-neurons and behavioral sensitivity to their respective agonists, capsaicin and mustard oil. Because functional interactions between TRPV1, TRPA1 and nAChR channels are reported in DRG neurons [31,49,50], we explored a possible interaction between the increase in nAChR subunits and TRP gene expression using ART-OE x TRPV1^−/−^/TRPA1^−/−^ hybrid mice. Results show that even in the absence of functional TRPV1 and TRPA1, Artn overexpression in skin increased transcription of mRNAs encoding α3 and β4 to levels equivalent to those in ART-OE mice. Therefore, the elevation in nAChRs is not a compensatory response to the increase in TRPV1 and TRPA1 in GFRα3 neurons and suggests a more direct role for Artn in nAChR regulation.

While we have focused on the role of peripheral nAChRs in the present study, changes in receptor density or function at central afferent terminals may also occur. In contrast to the pronociceptive role for nAChR signaling in peripheral terminals, the majority of studies using systemic or intrathecal delivery of nAChR agonists show spinal nAChR activation is antinociceptive [11,51-53]. Antinociception may result from changes in neurotransmitter release at presynaptic terminals and/or activation of descending noradrenergic and serotonergic systems [54,55]. Our behavioral and pharmacological data does not rule out the possibility of altered spinal presynaptic or descending signaling in response to long-term changes in Artn level in the periphery. Indeed, repeated injection of Artn was reported to block hyperalgesia and normalize neurochemical changes related to nerve injury in a rat model [56]. In addition, Artn (neublastin) is currently in Phase 2 clinical trial for treatment of sciatica-related pain [57]. In this preliminary study, some subjects reported a higher reduction in pain compared to placebo treated subjects. However, several subjects also reported mild to moderate adverse events that included headache, feeling hot, generalized pruritus and burning sensations. These events may result from the known effects of Artn on TRPV1 and TRPA1 expression and perhaps, as indicated by results of this study, on nAChR signaling as well.

## Conclusions

In chronic inflammatory conditions, increased expression of growth factors such as Artn is thought to alter gene expression and functional properties of sensory afferents. These changes can lead to increased neuron excitability that results in hypersensitivity. In mice that overexpress Artn in the skin, a significant increase in mRNAs encoding nAChR subunits α3, β3 and β4 and changes in nicotinic response properties in cutaneous afferents were observed. Thermal sensitivity evoked by Artn overexpression or by acute injection of Artn into footpad skin could be blocked by peripheral delivery of nicotinic antagonists. These findings indicate that the rise in Artn expression in inflamed tissue may regulate in a subunit specific manner, the expression of nAChR genes in primary afferents. This change in expression may contribute to changes in afferent properties that lead to hypersensitivity commonly associated with chronic inflammation.

## Methods

### Animals

Male and female artemin overexpresser (ART-OE) transgenic mice (described in Elitt et al. [10]) and strain and age-matched control littermates were used in these studies. ART-OE mice express a transgene in which the K14-keratin promoter drives expression of Artn in keratinocytes of the skin and oral cavity epithelium. For the CFA and Artn injection studies, 8–10 wk-old male, C57BL/6 J mice (Jackson Laboratories, Bar Harbor, ME) were used. Animals were housed in an American Association for the Accreditation of Laboratory Animal Care-accredited facility in a temperature and humidity controlled room on a 12 h light/12 h dark cycle with food and water provided *ad libitum*. Procedures were carried out in accordance with the guidelines of the University of Pittsburgh Institutional Animal Care and Use Committee.

### Chemicals and drugs

Rat Artn was generously provided by Biogen Idec (Weston, MA). Allyl isothiocyanate (AITC), capsaicin (CAP), cytisine, HC-030031, nicotine, hexamethonium, mecamylamine, methyllycaconitine (MLA) were purchased from Sigma-Aldrich (St. Louis, MO). Compound A-967079 was a generously supplied by AbbVie (North Chicago, IL). Artn was dissolved in saline at 200 ng/ml and 20 μl injected into footpad skin. For *in vivo* use mecamylamine and hexamethonium were dissolved in saline and 10 μl injected in footpad skin at 1 mg/kg body weight. These doses were chosen based on previous studies [6,58,59]. For *in vitro* studies mecamylamine was used at 50 μM and nicotine was used at concentrations between 1 μM and 3 mM. The α7 nAChR subunit antagonist MLA was dissolved in saline and used at a concentration of 20 nM. The TRPA1 antagonist HC-030031 was dissolved in DMSO at a concentration of 10 mM and diluted in bath solution to a final concentration of 10 μM based on results of prior studies [60]. The α3β4 nAChR subunit agonist cytisine was dissolved in normal bath solution and applied at a concentration of 100 μM [61]. The TRPV1 selective agonist capsaicin was dissolved in ethanol at a concentration of 10 mM and diluted in bath solution to a final concentration of 500 nM [62].

### Genome wide expression analysis

Mouse WG-6 Expression BeadChips (Illumina, San Diego, CA) were used to compare genes expressed in trigeminal ganglia of wildtype (C3HB6 hybrids) and ART-OE mice (n = 3 per group). In vitro transcription labeling, hybridization and data analysis was carried out by the University of Pittsburgh Genomics and Proteomics Core Laboratories (http://www.genetics.pitt.edu/).

### Reverse transcriptase real time PCR analysis

1 μg of RNA isolated from either pooled trigeminal or L2-L4 DRG was DNase-treated (Invitrogen Corporation, Carlsbad, CA), reverse-transcribed using Superscript II (Invitrogen Corporation, Carlsbad, CA) and analyzed using SYBR green PCR following manufacturers protocols. PCR primers (Table 5) were designed using MacVector software or software provided by Integrated DNA Technologies (Coralville, Iowa). SYBR green-PCR amplification reactions were performed on an Applied Biosystems 7000 real-time thermal cycler controlled by Prism 7000 SDS software (Applied Biosystems, Foster City, CA). Duplicate threshold cycle (C_t_) values were used as a measure of initial template concentration. Relative fold change in RNA was calculated using the ΔΔC_t_ method using glyceraldehyde-3-phosphate dehydrogenase (Gapdh) as a reference standard. Statistical significance between calculated ΔΔCt values determined using Student’s *t*-test with significance set a p < 0.05.

**Table 5:** List of PCR primers used in this study

**Gene**	**Primer pair (5′→3′)**
CHRNA2	GTGCCCAACACTTCCGATGT; CCAGCGCAGCTTGTAGTCAT
CHRNA3	TCCTGTCATCATCCAGTTTGAGG; TCATTCCAGATTTGCTTCAGCC
CHRNA4	CGCTTTGGCTTGTCGATTGC; AGTTTGTAGTCATGCCACTCCT
CHRNA5	GAAGGGGCCAGTACGAAAACA; AGCCGAATTTCATGGAGCAAT
CHRNA6	TAAAGGCAGTACAGGCTGTGA; AAAATGCACCGTGACGGGAT
CHRNA7	CACATTCCACACCAACGTCTT; AAAAGGGAACCAGCGTACATC
CHRNA9	GGAACCAGGTGGACATATTCAAT; GCAGCCGTAGGAGATGACG
CHRNB2	GAGGTGAAGCACTTCCCATTT; GCCACATCGCTTTTGAGCAC
CHRNB3	CAAAGGGGATGAAGGGCAAC; AAAGAGGGTGTAAAACAGGGGC
CHRNB4	TGGATGATCTCCTGAACAAAACC; CAGGCGGTAGTCAGTCCATTC
TRPV1	TTCCTGCAGAAGAGCAAGAAGC; CCCATTGTGCAGATTGAGCAT
TRPA1	GCAGGTGGAACTTCATACCAACT; CACTTTGCGTAAGTACCAGAGTGG
GAPDH	ATGTGTCCGTCGTGGATCTGA; GCTGTTGAAGTCGCAGGAGACA

### Tissue immunolabeling

Overlap of GFRα3 receptor and nAChRα3 expression in trigeminal ganglia of WT and ART-OE mice was detected using immunolabeling with tyramide signal amplification (Perkin Elmer, Waltham, MA). Ganglia from animals (n = 3 mice per group) perfused with 4% paraformaldehyde (PFA) made in 0.1 M phosphate buffer (PB) were post-fixed 2 h in 4% PFA at room temperature (RT). Ganglia were cryoprotected in 25% sucrose in 0.1 M PB overnight at 4°C, embedded in OCT compound (ThermoFisher Scientific) and sectioned at 20 μm thickness on a cryostat. Sections were washed in PB and incubated in rabbit anti-nAChRα3 (1:100, Santa Cruz Biotechnology, CA) in phosphate buffered saline (PBS) and 0.25% Triton X-100 at RT. Incubation times ranged from 8 h to overnight. Sections were washed, treated with 0.01% hydrogen peroxide solution in PBS for 30 min, washed and incubated 1 h at RT in horse radish peroxidase conjugated donkey anti-rabbit IgG (1:1000; Jackson ImmunoResearch, West Grove, PA). Sections were washed, incubated in rhodamine-conjugated AMP reagent (diluted 1:50 with kit reagent) for 10–20 minutes. Sections were then washed, incubated in goat anti-GFRα3 (R&D Systems, CA) 6 h-18 h, washed and then incubated in donkey anti-goat Cy2 (1:500; Jackson ImmunoResearch). Sections were washed, coverslipped in antifade solution (90% glycerol containing n-propyl gallate; see http://www.jacksonimmuno.com/technical/anti-fade.asp) and images captured using a digital camera attached to a Leica DM4000B fluorescence microscope (Leica, Wetzlar, Germany). Images captured from 5 non-overlapping sections of ganglia from 3 WT and 3 ART-OE mice were analyzed to determine the percentage of GFRα3-positive neurons that also exhibited staining for nAChRα3. All tissue processing, antibody labeling, image capture and processing were done in parallel. Statistical analysis of the data were done using Student’s t test with significance set at p < 0.05.

### Behavioral testing

Behavioral measures were made using a plantar testing apparatus (IITC, Woodland Hills, CA) in a blinded manner in the University of Pittsburgh Rodent Behavior Analysis Core. Animals were acclimatized for 1 h in the thermal testing apparatus for 2 days prior to testing. At 30 min following Artn or drug injection animals were placed individually in a small plexiglass enclosure with a glass floor. A focused light beam was applied to the plantar surface of the back feet. The mean of three measures of withdrawal latencies was determined at each timepoint tested. Data were analyzed using GraphPad Prism 4 software (San Diego, CA) and are presented as the mean ± SEM. Statistical analyses for differences over time in multiple groups were performed using two-way ANOVA followed by the Holm-Sidak post hoc test with significance set at p < 0.05.

### CFA inflammation

Mice were anesthetized with isoflurane and 20 μl of complete Freund’s adjuvant (CFA) emulsion was injected into the plantar surface of the hindpaw. All mice showed substantial edema after CFA injection. At day 0, 1 d and 3 d post-injection mice were deeply anesthetized and perfused transcardially with cold 0.9% saline. Paw skin and L3-L5 DRG were collected on dry ice and immediately processed for RNA isolation using Trizol reagent and RNeasy columns (Qiagen Inc., Valencia, CA), respectively. RNA was reconstituted in RNase-free water and processed for real time-PCR analysis.

### Patch clamp electrophysiology

DRG neurons that innervate the hind paw glabrous skin of WT and ART-OE mice were retrogradely labeled with 1,1′-Dioctadecyl-3,3,3′,3′-tetramethylindocarbocyanine perchlorate (DiI). L4 and L5 DRGs were enzymatically treated, mechanically dissociated and cultured as in [10]. Neurons were analyzed at 2–24 hours after dissociation. Voltage-clamp data were acquired with conventional whole cell patch clamp techniques with an Axopatch 200B amplifier (Molecular Devices, Sunnyvale CA) controlled with a PC running pClamp Software (V 10.3, Molecular Devices). Current traces were sampled at 5–10 kHz and filtered at 1–2 kHz. Patch electrodes were pulled from borosilicate glass (WPI, Sarasota FL) on a horizontal puller (Sutter Inst. Novato CA), and were 2–3 MΩ when filled with an electrode solution containing (in mM): K-methanesulfonate 110, KCl 30, NaCl 5, CaCl_2_ 1, MgCl_2_ 2, HEPES 10, EGTA 11, Mg-ATP 2, Li-GTP 1; pH 7.2 (adjusted with Tris-base), 310 mOsm (adjusted with sucrose). Currents were recorded in a bath solution containing (in mM): KCl 3, NaCl 130, CaCl_2_ 2.5, MgCl_2_ 0.6, HEPES 10, glucose 10; pH 7.4 (adjusted with Tris-base), 325 mOsm (adjusted with sucrose). The junction potential associated with all test solutions was measured and were all less than 5 mV, and therefore, junction potential was not corrected. Series resistance compensation was >80%. Whole-cell capacitance and series resistance were compensated with amplifier circuitry. Neurons were held at −60 mV and nicotine, cytisine, CAP and AITC were applied with a piezo- driven perfusion system (Warner Instruments, Hamden CT). Subpopulations of DRG neurons were defined by cell body size, binding of the plant lectin IB4 and responsiveness to CAP.

Current data were analyzed with pClamp software in combination with SigmaPlot (Systat, Chicago, IL). Current density was determined by dividing peak-evoked current by membrane capacitance (as determined with a 5 mV voltage step prior to compensation). Concentration-response data were fitted with a Hill equation to generate estimates of peak current (efficacy), the concentration 50% of peak (EC50, potency), as well as the Hill coefficient.

### Measure of acetylcholine

Footpad skin from C57BL/6 J mice injected 3 days prior with complete Freund’s adjuvant was immediately homogenized in PBS using a polytron (Kinematica AG). Equivalent amounts of supernatant (based on initial tissue weight) were assayed using the Amplex Red Acetylcholine/Acetlycholinesterase assay kit (Life Technologies, Molecular Probes). Fluorescence intensity was measured using a multiwell fluorescent plate reader (Gemini XPS; Molecular Devices, Sunnyvale, CA; excitation wavelength 560 nm and emission 590 nm) and μg/ml calculated using a standard curve.

## Competing interests

The authors declare that they have no competing interests.

## Authors’ contributions

KMA: study conception and design, acquisition of data, analysis and interpretation of data, writing of manuscript. XLZ: study design, acquisition of data, analysis and interpretation of data. CD: acquisition of data, analysis and interpretation of data. ESS: study design, acquisition of data, analysis and interpretation of data. CY: acquisition of data, analysis and interpretation of data. BMD: study design, acquisition of data, analysis and interpretation of data. MSG: study conception and design, acquisition of data, analysis and interpretation of data, writing of manuscript. All authors read and approved the final manuscript.
